# SOCIO-EMOTIONAL DEVELOPMENT ACROSS ADULTHOOD AND AGING

**DOI:** 10.1093/geroni/igae098.2221

**Published:** 2024-12-31

**Authors:** Urmimala Ghose, Denis Gerstorf, Jutta Heckhausen

**Affiliations:** Chair; Co-Chair; Discussant

## Abstract

Understanding how social relations evolve across adulthood, thereby shaping well-being in later life is a longstanding question in lifespan developmental research. The presented studies offer novel insights, utilizing innovative methodologies, diverse time scales, and varied samples. Chopik combined qualitative data from the Harvard Student Study Class of 1964 with machine learning techniques, reporting a larger and closer friends’ circle during freshman year to be associated with more positive life reflections, enhanced relationships, and reduced mental illness fifty years later. Chopik also explored historical changes in these associations using a novel cohort sequential design. Fiori et al. analyzed hierarchical network mapping and ecological momentary assessment data from the Stress and Well-being in Everyday Life study. Contrary to common theoretical expectations, they found that only interactions within the inner circle of social networks declined with age, and increased positivity in interactions extended beyond the inner circle. Hülür et al. conducted an event-contingent experience sampling study on older adults revealing that interactions with more close social partners were linked to higher well-being across communication modes. Digital text-based communication was identified as a compensatory mechanism for individuals with smaller social networks. Ghose et al. utilized a unique synthetic cohort matching method with Health and Retirement Study panel data, illustrating distinct well-being trajectories in older adults undergoing marital transitions compared to their never/continuously-married peers. Their results suggested more pronounced initial increase and subsequent decrease in depressive symptoms following spousal bereavement than divorce, with social strain identified as an additional risk factor in bereavement.

